# Effectiveness Of Digital Therapeutics On Chronic Pain In Older People: A Systematic Review And Meta-Analysis

**DOI:** 10.1093/geroni/igaf122.3213

**Published:** 2025-12-31

**Authors:** Jeongeun Lee, Seyeon Park

**Affiliations:** Chungnam National University College of Nursing, Daejeon, Republic of Korea; Chungnam National University College of Nursing, Daejeon, Republic of Korea

## Abstract

**Background:**

Chronic pain in older people is a global health issue, reducing quality of life and increasing healthcare costs. Traditional treatments have limited long-term effectiveness due to side effects and dependency risks, driving interest in non-pharmacological approaches like digital therapeutics (DTx), which use technologies such as mobile apps, VR, AI, and digital CBT to modulate pain and promote neuroplasticity.

**Purpose:**

This objective of this study is to evaluate the effectiveness of DTx in reducing chronic pain in older people through a systematic review and meta-analysis to assess the effect size.

**Methods:**

A systematic review and meta-analysis following PRISMA 2020 guidelines. A comprehensive search of major E-databases was conducted to identify studies examining the effects of digital therapeutics on chronic pain management. A total of 106 studies were initially retrieved, and 6 studies that met the inclusion criteria were selected for analysis. The effect size of Pain reduction was analyzed using Standardized Mean Difference (SMD) and a random-effects model to address heterogeneity.

**Results:**

The meta-analysis revealed that digital therapeutics had a statistically significant small effect on reducing chronic pain in older people, with an SMD of -0.31 (95% CI: -0.57, -0.05, p < 0.05). Additionally, all 6 included studies were recent (published between 2000-2004), and no studies had a high risk of bias.

**Conclusion:**

The findings suggest that DTx can be an effective non-pharmacological strategy for pain management in older adults, but further large-scale and long-term studies are needed to confirm their sustained effectiveness and broader applicability.

